# Recurrent Intrathoracic Liposarcoma: A Case Report and a Comprehensive Literature Review of a Rare Clinical Entity

**DOI:** 10.7759/cureus.70241

**Published:** 2024-09-26

**Authors:** Vasileios Leivaditis, Manfred Dahm, Athanasios Papatriantafyllou, Hans-Georg Keul, Lydia Kohl, Hans-Joachim Schäfers

**Affiliations:** 1 Department of Cardiothoracic and Vascular Surgery, Westpfalz-Klinikum, Kaiserslautern, DEU; 2 Department of Pathology, Westpfalz-Klinikum, Kaiserslautern, DEU

**Keywords:** adjuvant therapy, dedifferentiated liposarcoma (ddlps), long-term follow-up, pleural liposarcoma, recurrent intrathoracic liposarcoma, surgical resection

## Abstract

Liposarcomas (LPSs) are rare malignant tumors of adipocytic origin, primarily occurring in the extremities and retroperitoneum, with thoracic involvement being exceptionally rare. This case report details the surgical management and outcomes of a recurrent intrathoracic LPS in a 65-year-old male with a history of previous mediastinal tumor resection. CT imaging revealed a recurrent tumor extending into the left pleura. The patient underwent a posterolateral thoracotomy for complete tumor excision and limited replacement of the descending aorta. Postoperative recovery was smooth, and histology confirmed dedifferentiated LPS (G2) with areas of highly differentiated LPS. LPSs encompass a heterogeneous group of tumors with various subtypes, including atypical lipomatous tumor/well-differentiated liposarcoma (ALT/WDLPS), dedifferentiated liposarcoma (DDLPS), myxoid liposarcoma (MLPS), and pleomorphic liposarcoma (PLPS). Treatment primarily involves complete surgical resection, while the roles of radiotherapy and chemotherapy remain debated. Immunotherapy shows potential benefits, particularly for DDLPS patients expressing PD-L1. Prognosis varies significantly by subtype, with DDLPS and PLPS associated with poorer outcomes compared to MLPS and ALT/WDLPS. Long-term follow-up is crucial for managing LPSs due to their high recurrence rate. This case highlights the effectiveness of surgical intervention in recurrent intrathoracic LPSs and underlines the need for continued research into adjuvant therapies to improve patient outcomes.

## Introduction

Liposarcomas (LPSs) are one of the most prevalent sarcomas in adults [1-3]. As rare malignant tumors of adipocytic origin, LPSs typically occur in the extremities and retroperitoneum [4]. LPSs in the pleura or thoracic cavity are particularly rare, with few cases documented in the literature [2,3,5-10].

LPSs account for 15%-25% of all soft tissue sarcomas, making them the most common sarcoma in adults. The World Health Organization classifies LPSs into five subtypes: atypical lipomatous tumor/well-differentiated liposarcoma (ALT/WDLPS), dedifferentiated liposarcoma (DDLPS), myxoid liposarcoma (MLPS), pleomorphic liposarcoma (PLPS), and myxoid pleomorphic liposarcoma (MPLPS) [4,11].

ALT/WDLPS typically occurs in the deep soft tissues of the proximal extremities and trunk, frequently involving the retroperitoneum and paratesticular area. Rare sites include the head and neck, mediastinum, distal extremities, and skin [4,12]. DDLPS often arises in the retroperitoneum and spermatic cord, but can also appear in the mediastinum, head and neck, and trunk. MLPS generally presents in the deep soft tissues of the extremities and thigh, and very rarely in the subcutis or retroperitoneum. PLPS most commonly affects the extremities, with less frequent involvement of the trunk wall, retroperitoneum, and spermatic cord. It is rarely found in the mediastinum, heart, pleura, and breast. MPLPS is frequently seen in the mediastinum, with other reported sites including the thigh, head, neck, perineum, abdomen, and back [1,4,12].

Despite the rarity of pleural involvement, LPSs can recur, necessitating vigilant long-term follow-up [8]. This case report discusses the surgical management of a recurrent intrathoracic LPS, detailing the challenges encountered and the outcomes achieved.

## Case presentation

Patient history

**Figure 1:**
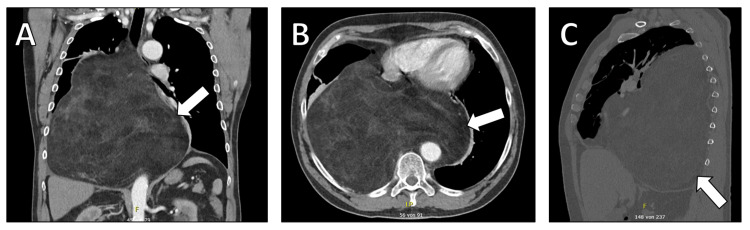
Computed tomography imaging demonstrating the liposarcoma (arrow) resected five years ago. (A) Coronal view showing the size and position of the original tumor within the thoracic cavity. (B) Transverse view highlighting the tumor's relationship to the surrounding anatomical structures. (C) Sagittal view providing a detailed perspective on the tumor's depth and its proximity to vital organs.

A 65-year-old male with a history of arterial hypertension presented with dyspnea. The patient had a significant surgical history of mediastinal tumor resection from the right pleura five years prior, with histological confirmation of an LPS (Figure 1). The patient did not however demonstrate consistent follow-up after the operation.

Clinical findings and diagnostic assessment

**Figure 2:**
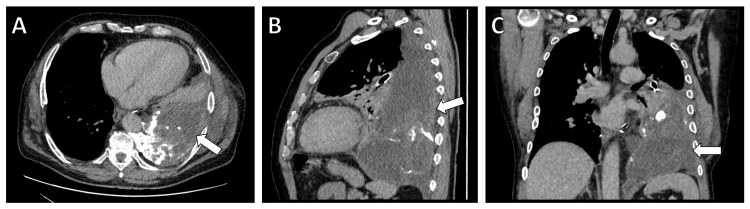
Computed tomography imaging demonstrating the recurrence of the liposarcoma (arrow) in the left pleural cavity. (A) Transverse view illustrating the extent of the recurrent tumor within the left pleural cavity. (B) Sagittal view showing the tumor's infiltration into adjacent structures. (C) Coronal view detailing the spread and impact of the recurrent tumor on the pleural cavity.

**Figure 3:**
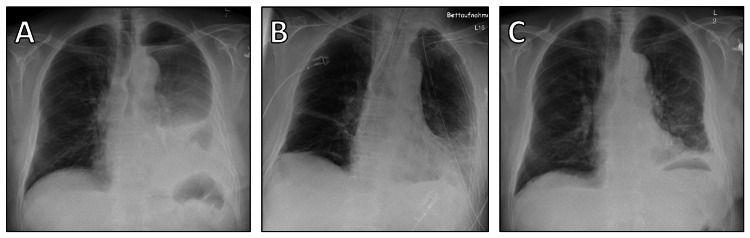
Chest X-rays at different stages of treatment. (A) Preoperative image displaying the recurrent tumor's impact on lung fields and mediastinal structures. (B) Immediate postoperative image indicating the successful resection and the initial postoperative state of the thoracic cavity. (C) Image at discharge showing the recovery progress and the state of the thoracic cavity post-treatment.

On presentation, the patient exhibited symptoms of dyspnea without other systemic symptoms. Computed tomography (CT) revealed a recurrent tumor with extension into the left pleura, suggesting recurrence of the previously resected LPS. CT imaging demonstrated a sizable mass extending into the left pleura (Figures 2, 3A). Preoperative assessments included routine blood tests, ECG, and pulmonary function tests, all of which were within acceptable limits for surgery.

Therapeutic intervention

**Figure 4:**
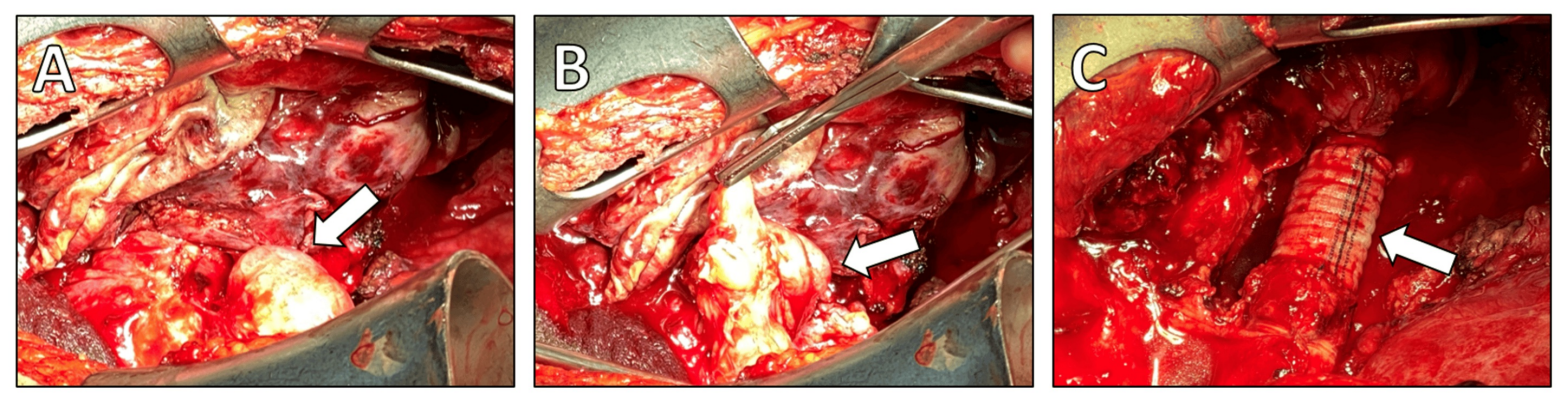
Intraoperative access to the tumor. (A) Identification of the tumor (arrow) in the left pleural cavity, illustrating its location and size relative to the pleura. (B) Resection of the tumor (arrow), demonstrating the surgical process and the extent of the excised tissue. (C) The descending aorta after local segmental replacement (arrow), showing the repair performed due to local infiltration by the tumor.

**Figure 5:**
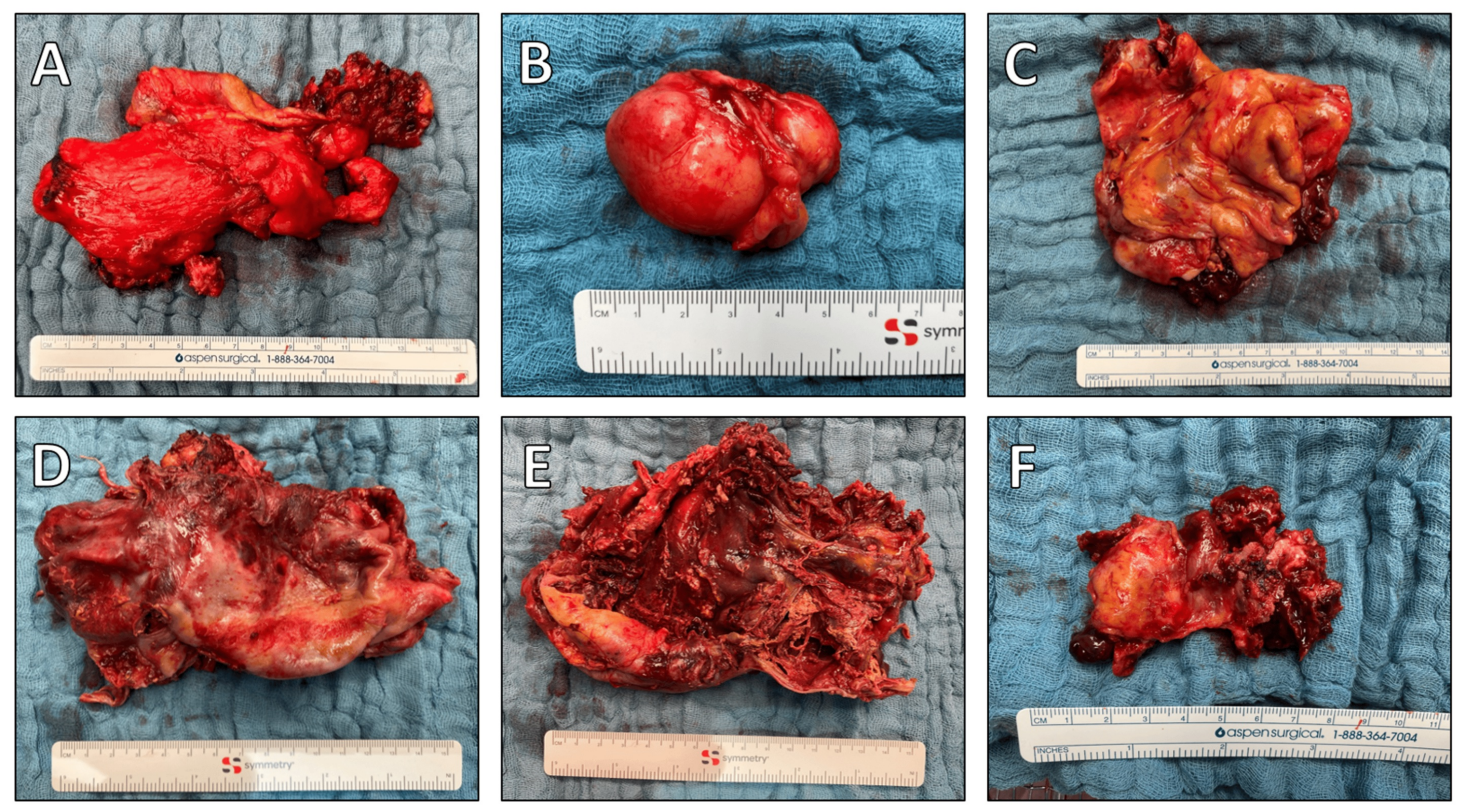
Segments of the resected tumor. Detailed view of the different sections of the tumor removed during surgery, highlighting the varied morphology and size of the excised segments.

Given the symptomatic nature of the recurrence and potential prognostic implications, surgical intervention was indicated. The left hemithorax was accessed via a posterolateral thoracotomy. Intraoperatively, extensive inflammatory adhesions were dissected to reveal the tumor originating from the mediastinum with local infiltration of the descending aorta (Figures 4A, 4B). Complete excision of the tumor was achieved (Figure 5), followed by a limited replacement of the locally infiltrated descending aorta segment (Figure 4C).

Postoperative course

The patient tolerated the procedure well and was transferred to the intensive care unit (ICU) in stable condition. The postoperative course was uncomplicated; the patient was extubated on the day of surgery and transferred to the normal ward on the first postoperative day (Figure 3B). Chest drains were removed sequentially on the second and third postoperative days.

**Figure 6:**
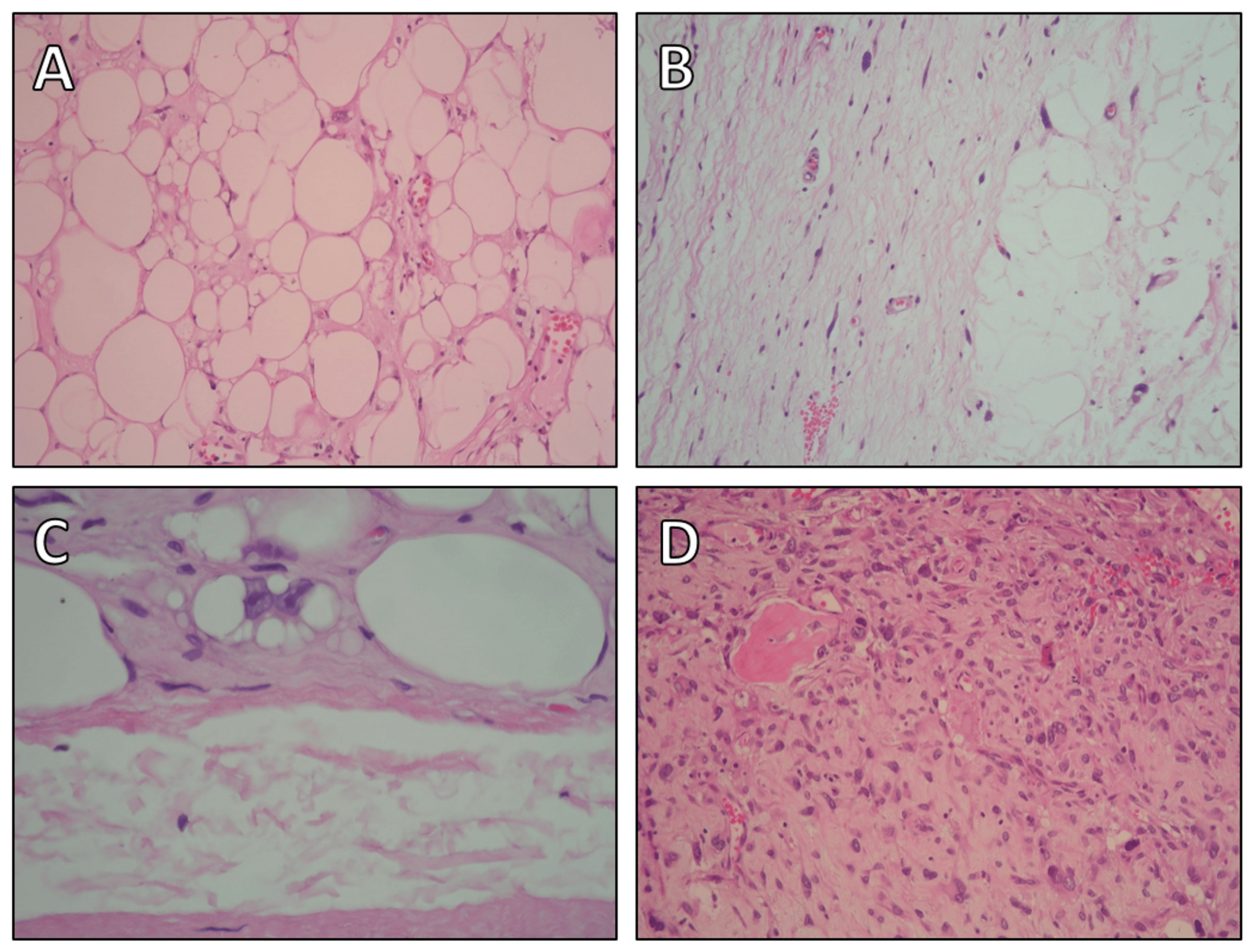
Histological images of the resected liposarcoma. (A) Section of a highly differentiated liposarcoma with sclerotic areas, displaying varying sizes of fat cells. (B) Another section of a highly differentiated liposarcoma with sclerotic areas, highlighting an enlarged nucleus with hyperchromasia. (C) Typical lipoblast with a double nucleus and small fat vacuoles within the cytoplasm. (D) Details of the dedifferentiated part of the liposarcoma, showing pronounced nuclear polymorphism and nonrecognizable fat cells. Additionally, a small osteoid formation is evident, indicating osteosarcomatous differentiation.

Histological examination of the excised tumor revealed a dedifferentiated LPS (G2 according to FNCLCC) with areas of highly differentiated LPS and an osteosarcomatous heterologous component (Figure 6). The patient was discharged on the seventh postoperative day, clinically asymptomatic with unremarkable wound healing (Figure 3C).

Follow-up and outcomes

Post-discharge follow-up at one week and one month showed no recurrence of symptoms, and physical examinations revealed no pathological findings. The patient remained symptom-free and in good general health.

## Discussion

This case highlights the critical importance of long-term follow-up in patients with LPSs due to their high recurrence rate. Surgical resection remains a pivotal treatment approach for recurrent intrathoracic LPSs, as evidenced by the successful outcome in this case, despite the tumor's complex localization and involvement of the descending aorta. The patient's smooth postoperative recovery and symptom-free follow-up further demonstrate the effectiveness of surgical intervention in managing recurrent LPSs.

Soft tissue sarcomas, originating from mesenchymal cells, encompass nearly 50 histological subtypes, with LPSs accounting for 20-30% of these tumors in adults [3]. First described by Virchow in 1860, LPSs most commonly arise between the fifth and seventh decades of life, with an average onset age of 43-49 years, and are rare in children and young adults [3,13]. The retroperitoneum is the most frequent site of LPSs, followed by the extremities, with occurrences in the gastrointestinal tract and thoracic cavity being notably rare [2]. The FNCLCC grading system, introduced by the French Federation of Cancer Centers Sarcoma Group in 1984, assesses the malignancy of these tumors (Tables 1, 2) [10,14]. In our case, an intermediate-grade (Grade 2) LPS was histologically diagnosed.

**Table 1:** The French Federation of Cancer Centers Sarcoma Group (FNCLCC) grading system for soft tissue sarcomas. The FNCLCC grading system is used to assess the malignancy of soft tissue sarcomas. The grading system is based on three parameters: tumor differentiation, mitotic count, and necrosis. Each parameter is scored, and the total score determines the grade of the sarcoma.

Parameter	Score	Description
Tumor Differentiation		
Sarcomas closely resembling normal adult mesenchymal tissue	1	Example: Well-differentiated liposarcoma
Sarcomas with specific histologic typing	2	Example: Myxoid liposarcoma
Sarcomas with undifferentiated morphology	3	Example: Pleomorphic sarcoma, sarcomas of doubtful type, and synovial sarcomas
Mitotic Count (per 10 high-power fields)		
0-9 mitoses	1	Low mitotic activity
10-19 mitoses	2	Intermediate mitotic activity
≥20 mitoses	3	High mitotic activity
Necrosis		
No necrosis	0	Absence of necrotic areas
<50% necrosis	1	Partial necrosis
≥50% necrosis	2	Extensive necrosis

**Table 2:** Total score and grade according to the FNCLCC grading system. FNCLCC: French Federation of Cancer Centers Sarcoma Group

Total Score	Grade	Description	Interpretation/Prognosis
2-3	Grade 1	Low grade	Low malignancy potential, better prognosis.
4-5	Grade 2	Intermediate grade	Intermediate malignancy potential.
6-8	Grade 3	High grade	High malignancy potential, worse prognosis.

Among malignant pleural neoplasms, pleural LPSs are exceptionally rare, with only 1% of all LPSs occurring in the thorax [12,15]. Primary intrathoracic sarcomas are uncommon, with most cases being metastatic and originating from various chest tissues, including the mediastinum, pleura, pulmonary artery, or lung [3].

Symptoms and clinical characteristics

Thoracic LPSs typically exhibit an expansile growth pattern rather than an infiltrative one, often presenting with nonspecific symptoms despite their large size [2]. Pain can occur if the tumor invades nerves in the chest wall. Mediastinal LPSs, which account for less than 1% of all mediastinal tumors, often grow slowly and remain asymptomatic until they reach a size that compresses adjacent structures [6]. Symptoms of large mediastinal lipomas and well-differentiated LPSs can include superior vena cava syndrome, Horner’s syndrome, dysphagia, dyspnea, cough, spinal nerve paralysis, tachycardia, and heart failure [11].

The clinical course of LPS patients is largely determined by the tumor location and size. According to Kiełbowski et al., 16% of patients are asymptomatic at diagnosis, with tumors discovered incidentally. Over time, tumor growth leads to compression of nearby structures, causing symptoms. Dyspnea is the most common symptom (50%), followed by chest pain (29%) [3]. Similarly, Baheti et al. found that among patients with intrathoracic synovial sarcoma, 21% were asymptomatic, while 49% experienced chest pain and 19% had dyspnea [16]. In a study by Xiao et al., 44% of retroperitoneal LPS patients were asymptomatic, while 32% reported abdominal distortion and 28% reported pain [17]. Our patient also presented with dyspnea, with a CT scan revealing compression of the left lower lobe.

Diagnostic imaging

CT imaging is the most cost-effective and efficient method for diagnosing LPSs [4]. The appearance of LPSs on CT scans can vary significantly, ranging from discrete solid masses to highly vascular or fat-containing lesions. A recent advancement in this field is the use of four-dimensional computed tomography (4DCT) to assess the adhesion of LPSs to adjacent organs. Unlike magnetic resonance imaging (MRI), which requires deep inspiratory breath holds, 4DCT does not, making it more convenient for patients [2,18]. Differentiating between a lipoma and a lipoma-like well-differentiated LPS can be challenging, especially with large tumors. Both types typically show homogeneous fat attenuation on CT scans [4], but well-differentiated LPSs often have thicker, more irregular, or nodular fibrous septa compared to lipomas [19]. MRI is advantageous for evaluating soft tissue involvement, boasting an 83% accuracy rate in diagnosing well-circumscribed LPSs [11]. Angiography plays a crucial role in detecting the blood vessels supplying the tumor and understanding their relationship with nearby organs. This technique is particularly useful when CT scans do not conclusively determine the tumor's location [2,18].

The significant inhomogeneity of the tumor posed a notable diagnostic challenge in this case. The LPS exhibited a mix of well-differentiated and dedifferentiated areas, along with regions showing osteosarcomatous differentiation. This variability in tissue composition made it difficult to differentiate the tumor from other thoracic masses, both radiologically and histologically. CT imaging revealed a mix of soft tissue and fatty components, complicating the interpretation, while the heterogeneous nature of the mass meant that biopsy samples may not always represent the more aggressive areas of the tumor, increasing the risk of misdiagnosis. This variability also has implications for treatment, as different tissue components may respond differently to therapies. Therefore, a thorough preoperative evaluation and a multidisciplinary approach are essential for accurate diagnosis and optimal treatment planning.

Histological subtypes

ALT-WDLPS

ALT-WDLPS is the most common subtype of LPSs, characterized by a simple genomic profile with a 12q14-15 amplification involving the MDM2 gene [1,11]. ALT-WDLPS is a mesenchymal neoplasm that can consist entirely or partially of mature adipocytic cells, usually showing nuclear atypia in stromal cells [1,4]. This subtype is divided into three categories: (i) lipoma-like, (ii) sclerosing, and (iii) inflammatory [4,20]. Diagnosis does not require the presence of lipoblasts, and immunohistochemistry for CDK4 and MDM2 is useful in distinguishing ALT-WDLPS from benign lipomatous lesions [4]. ALT-WDLPS does not metastasize unless it has a dedifferentiated component, allowing for curative wide resection in limbs or trunk wall cases. ALT and WDLPS are synonymous, describing lesions identical in morphology, genetics, and biological potential [1,20].

ALT-WDLPS accounts for 30-40% of all LPSs and predominantly affects men in their sixth and seventh decades [1,3]. These tumors are most common in the deep soft tissues of the limbs (especially the thigh), retroperitoneum, paratesticular area, and groin, with rare occurrences in the mediastinum, subcutaneous tissue, and other parenchymal sites [4,20]. While ALT-WDLPS tumors do not metastasize, their local recurrence rate depends on their location. Extremity tumors have significantly lower recurrence rates compared to retroperitoneal tumors, which almost always recur and are often fatal. The risk of dedifferentiation increases over time, with more than 20% of retroperitoneal tumors and less than 5% of extremity tumors undergoing dedifferentiation [3,20]. Overall mortality ranges from 0% for limb tumors to over 80% for retroperitoneal tumors with long-term follow-up [1,19].

DDLPS

DDLPS, a term introduced by Evans in 1979, describes LPSs containing ALT-WDLPS juxtaposed with high-grade non-lipogenic sarcoma, often resembling high-grade pleomorphic sarcoma or fibrosarcoma [21]. DDLPS accounts for 18% of LPSs [7]. DDLPS commonly occurs in late adulthood without sex predilection, predominantly in the retroperitoneum (over 80% of cases), extremities, spermatic cord, and other internal trunk sites. It is rare in the head, neck, and subcutaneous tissues [19].

Initially believed to arise from ALT-WDLPS over several years, it is now recognized that most DDLPS cases arise de novo and are identified during the initial excision [10]. DDLPS can be diagnosed without the presence of ALT-WDLPS areas due to its specific genomic profile, although inappropriate sampling or the disappearance of ALT-WDLPS components might obscure their presence. Genomic abnormalities indicate that DDLPS is a malignant adipocytic tumor progressing from ALT-WDLPS to non-lipogenic sarcoma of varying grades. Approximately 90% of DDLPS cases arise de novo, while 10% occur in recurrence, with a higher risk in deep-seated tumors like those in the retroperitoneum [1,20]. DDLPS demonstrates histological transitions from WDLPS to non-lipogenic spindle or pleomorphic sarcoma, mimicking high-grade fibrosarcoma or undifferentiated pleomorphic sarcoma [1,4]. Immunohistochemistry for MDM2 and CDK4 is crucial for diagnosing DDLPS, with reported sensitivities of 95% and 92% and specificities of 81% and 95%, respectively [1].

In our case of recurrent LPS, histology showed predominately a dedifferentiated LPS with some areas of highly differentiated LPS. Prognosis is dominated by local recurrences (40-60%), particularly in the retroperitoneum, with a low metastatic potential (15-20%) [1]. Retroperitoneal cases nearly always recur over 10-20 years, with overall mortality ranging from 30% to 40% at five years. Anatomic location is the most critical prognostic factor, with retroperitoneal tumors having a poor prognosis. Recent studies suggest that the grade and extent of dedifferentiation are predictors for event-free survival in retroperitoneal DDLPS [1,19]. DDLPS has a poorer prognosis than WDLPS in cases of intrathoracic LPS, but its clinicopathological characteristics remain unclear due to its rarity, especially when originating from the mediastinum [10].

PPLS

PPLS is the rarest subtype of LPSs, accounting for less than 15% of all cases [4]. Unlike other types, PPLS primarily develops de novo without ALT/WDL-like low-grade precursor lesions and lacks MDM2 amplification [7]. It has a complex genomic profile with numerous gains and losses, similar to those seen in poorly differentiated sarcomas and particularly in myxofibrosarcomas [1,4]. The most common histologic pattern resembles an undifferentiated pleomorphic sarcoma with giant lipoblasts.

PPLS typically grows slowly within the pleural cavity, often remaining asymptomatic for extended periods. Clinical manifestations are delayed and nonspecific, including symptoms such as cough, dyspnea, and chest pain, which result from the displacement and compression of adjacent structures. PPLS can also be an incidental finding in imaging studies [9]. According to Hornick et al., multivariate analysis identified age ≥60 years, central location, tumor size, and mitotic rate as independent predictors of adverse outcomes [5].

MLPS

MLPS consists of uniform, round-to-ovoid cells, formerly referred to as round cell LPSs, with variable numbers of small lipoblasts set in a myxoid stroma with branching capillary vasculature [1]. In the literature, up to 33% of cases in the pleural cavity have been identified as MLPS, making it one of the most common histologic types in this location [3]. Genetically, MLPS is defined by the presence of the recurrent translocation of DDIT3 (DNA Damage-Inducible Transcript 3) on chromosome 12, leading to the fusion of the DDIT3 (CHOP) gene on 12q13 and the FUS (TLS) gene on 16p11 [1,4]. MLPS exhibits distinct clinical characteristics compared to other soft tissue sarcoma subtypes. While MLPS can progress slowly, certain tumors may metastasize to atypical locations not commonly associated with other sarcomas. Unlike most soft tissue sarcomas, which primarily metastasize to the lungs, MLPS can also spread to extrapulmonary sites, including soft tissues and bones [22]. Survival rates for localized MLPS are generally higher than for other sarcomas, with five-year overall survival ranging from 78% to 91%. However, prognosis is significantly worse for patients with metastatic disease at diagnosis or tumors containing a round cell component, emphasizing the critical importance of thorough staging [4,22].

Treatment

Due to its rarity, there is no established treatment algorithm for this clinical entity. Surgery is however widely regarded as the most favorable option for the suitable candidates.

Surgical Treatment

Surgical resection is the primary and most effective treatment strategy for intrathoracic LPSs. Complete surgical resection is crucial, as leaving even a small fragment of the tumor or its capsule can worsen survival rates and increase the risk of recurrence [6]. The procedure involves removing the tumor along with a 1 cm margin of normal tissue or a major fascial barrier circumferentially. If the tumor encases a major neurovascular structure, resection may necessitate arterial reconstruction [23]. For minor lesions, the video-assisted thoracoscopic surgery technique is often successful. However, larger tumors typically require open surgeries, such as posterolateral or anterolateral thoracotomy, depending on the tumor's location and the surgeon's preference. Tumors in the anterior mediastinum, which may involve large vessels, can be particularly challenging. In some cases, extensive incisions like bilateral anterior thoracotomy, median sternotomy, or clamshell access are necessary for better exposure. For tumors infiltrating the posterior mediastinum, appropriate surgical access is vital [3,6,11].

Radiotherapy

The role of radiotherapy (RTH) is still debated. Some studies, like those by Lee et al., suggest that combining RTH with surgery can reduce recurrence in patients with histological subtypes other than WDLPS, although the results were not statistically significant [24]. A recent phase 3 clinical trial (STRASS) indicated potential benefits of preoperative RTH, warranting further investigation [25]. MLPS is particularly sensitive to radiation, which supports the use of preoperative radiotherapy for this subtype [3]. Adjuvant radiation therapy is recommended for patients with unresectable malignancies to improve local tumor control and reduce the risk of recurrence [3,13]. Adjuvant radiation is used to lower the risk of local recurrence in cases of high-grade DDLS of the extremity when the tumor exceeds 5 cm in diameter or following an R1 resection that cannot be improved without significant morbidity. Due to the relative chemoresistance of DDLS, systemic therapies are rarely used for localized cases [23].

Chemotherapy

Chemotherapy has been explored in the treatment of LPSs, with reports including drugs like ifosfamide, adriamycin, lipozonid, doxorubicin, docetaxel, and pazopanib; however, its role in the treatment of LPSs remains very limited. However, pazopanib is not approved for LPSs despite showing preclinical activity in DDLPS [3]. The evidence regarding chemotherapy's effectiveness in LPSs is mixed. While it does not consistently prolong overall survival, its efficacy may vary depending on the sarcoma's histological subtype. MLPS and PLPS are generally more responsive to chemotherapy than WDLPS and DDLPS [3,26].

Immunotherapy

The molecular landscape of LPSs indicates potential benefits from immunotherapy. For instance, programmed death-ligand-1 (PD-L1) expression is found in 31.5% of WDLPS and 51.3% of DDLPS cases, suggesting that immune checkpoint inhibitors (ICIs) like nivolumab and ipilimumab could be effective [27]. DDLPS patients, in particular, are considered potential candidates for ICI treatment. Ongoing clinical trials are evaluating other immunotherapies, such as CAR T cells and cytokine administration, to determine their efficacy in LPS treatment [19].

Prognosis

Patients with DDLPS or PLPS have a significantly worse prognosis and lower survival rates compared to those with MLPS or WDLPS. DDLPS, in particular, shows higher rates of metastasis (17%) and disease-related mortality (28%) compared to WDLPS [24]. Long-term and vigilant follow-up remains essential [8].

Regarding recurrent disease, due to the relative resistance of WDLPS/DDLPS to systemic therapy, surgical re-resection has been the standard approach for managing recurrent disease. However, resection of recurrent tumors is associated with higher complication rates, making careful patient selection crucial to identify those who may benefit from alternative treatments [23].

A key factor in the management of LPSs is strict adherence to postoperative follow-up, which plays a crucial role in detecting recurrences early. In our case, the patient did not demonstrate consistent follow-up after the initial operation, leading to delayed recognition of tumor recurrence. Given the high recurrence rates associated with LPSs, especially in cases involving dedifferentiated subtypes, regular monitoring is essential for timely intervention. Routine imaging and clinical assessments allow for early detection of recurrent or residual disease, which can improve outcomes through earlier resection or adjuvant therapy. Without proper follow-up, patients may present at a more advanced stage of recurrence, as seen in this case, which can complicate treatment and negatively affect prognosis. This underscores the critical need for structured, long-term follow-up protocols to optimize the management of LPSs and improve patient outcomes.

## Conclusions

This case highlights the importance of vigilant long-term follow-up and thorough management in patients with LPSs due to the high risk of recurrence. Surgical resection remains the cornerstone of treatment for recurrent intrathoracic LPSs, as demonstrated by the successful outcome in this case despite the tumor's complex involvement of the descending aorta. The patient's smooth postoperative recovery underlines the efficacy of surgical intervention. However, the variability in prognosis among different LPS subtypes necessitates continued research into adjuvant therapies, including radiotherapy, chemotherapy, and immunotherapy, to optimize treatment outcomes and improve patient survival.
